# A Case of a Patient With Spinal Muscular Atrophy With Dysphagia Who Acquired Vacuum Swallowing

**DOI:** 10.7759/cureus.53129

**Published:** 2024-01-28

**Authors:** Keishi Okamoto, Kenjiro Kunieda, Tomohisa Ohno, Mika Ogawa, Ichiro Fujishima

**Affiliations:** 1 Rehabilitation, Hamamatsu City Rehabilitation Hospital, Shizuoka, JPN; 2 Neurology, Gifu University Graduate School of Medicine, Gifu, JPN; 3 Dentistry, Hamamatsu City Rehabilitation Hospital, Shizuoka, JPN

**Keywords:** swallowing disorders, lower esophageal sphincter, high-resolution manometry, pharyngeal pressure, negative pressure in esophagus, dysphagia, neuromuscular disorders, vacuum swallowing

## Abstract

We present a case of acquired vacuum swallowing in a patient with spinal muscular atrophy associated with dysphagia. A 67-year-old male presented with spinal muscular atrophy. Even though he was able to eat orally, he required a long time to eat and faced difficulty while swallowing saliva, resulting in frequent spitting. Instructions regarding vacuum swallowing to eliminate pharyngeal residue were provided, and a reduction in meal duration and improved saliva swallowing were observed. High-resolution manometry revealed a significant increase in pharyngeal contractile integral and a significant decrease in esophageal pressure with vacuum swallowing, which enabled the passage of a bolus through the pharynx compared with non-vacuum swallowing. Furthermore, an increase in the lower esophageal sphincter pressure, reflecting diaphragmatic contraction, was also observed. Therefore, this case report elucidates that a patient with neuromuscular disorders could acquire vacuum swallowing with proper instructions.

## Introduction

Vacuum swallowing is an innovative method that improves bolus passage through the pharynx by creating negative pressure in the esophagus, particularly in patients with dysphagia due to lateral medullary syndrome (LMS) [1-3]. The voluntary contraction of inspiratory muscles during swallowing creates a strong negative pressure in the thoracic cavity. Consequently, a negative pressure is generated in the esophagus. Observation of the patient's body surface revealed a depression in the anterior trachea and supraclavicular fossa due to contraction of the sternocleidomastoid muscle which is the inspiratory muscle [4]. This technique facilitates the passage of the bolus from the pharynx to the esophagus by creating a pressure gradient that compensates for impaired pharyngeal contraction and the opening of the upper esophageal sphincter (UES) [1, 2].

The ability to reproduce vacuum swallowing in patients with dysphagia due to LMS has been demonstrated [4]. However, no study has confirmed the acquisition of vacuum swallowing in patients with neuromuscular diseases. Herein, we present the case of a patient with dysphagia due to spinal muscular atrophy (SMA) who acquired vacuum swallowing through instructions.

## Case presentation

A 67-year-old man presented with SMA. His medical history showed an increased susceptibility to falls from the age of 15. Genetic testing for SMA was performed eight years ago; however, the genetic mutations were not identified. Further, a neurologist clinically diagnosed SMA. Dysphagia developed approximately seven years ago, leading to gastrostomy placement six years ago. Balloon dilatation [5] was introduced five years ago to relieve the impaired UES opening. He was admitted to our hospital with a fracture in the right lateral condyle of the femur. Although he required complete assistance with the activities of daily living (ADL), his cognitive function was preserved.

Neurological examination revealed facial and lingual muscle atrophy and weakness. Physical examination and nutritional assessment revealed a height of 165 cm, weight of 56.1 kg, body mass index (BMI) of 20.61, serum albumin of 3.8 g/dl, geriatric nutritional risk index (GNRI) [6] of 95 (reflecting mild nutritional risk), and creatinine of 0.18 mg/dl. Dysphagia screening revealed the repetitive saliva swallowing test (RSST) [7, 8] as normal with seven counts/30 seconds and the Mann Assessment of Swallowing Ability (MASA) [9] score of 139 (dysphagia: moderate, aspiration: severe). According to the Food Intake LEVEL Scale (FILS) [10], he was rated level eight after consuming a dysphagia diet-4 [11] coupled with pre-meal balloon dilatation. However, he had pharyngeal residue and frequent wet swallowing, and it took over an hour to eat. In addition, swallowing saliva was difficult, and self-expectation was required.

On day 11 after admission, videofluoroscopy (VF) revealed oral dysfunction due to decreased palatal height and tongue strength, requiring compensatory cervical extension. The pharyngeal phase showed incomplete laryngeal elevation, reduced pharyngeal contraction, and pharyngeal residue owing to insufficient UES passage of the bolus. Immediate improvement was observed after balloon dilatation of the impaired UES. The devised plan included the creation of a palatal augmentation prosthesis (PAP) [12, 13] to address oropharyngeal concerns and coaching for vacuum swallowing to eliminate pharyngeal residue during speech therapy.

Vacuum swallowing was taught step-by-step from day 15 using the approach of Kunieda et al. [4] to create a strong negative pressure in the thoracic cavity, ideal timing of swallowing, and inspiratory effort. The patient was instructed to attempt to inhale with the mouth closed (inspiratory effort), such that there is no airflow into the airway. The anterior trachea and supraclavicular fossa were depressed, reflecting the creation of negative pressure in the thoracic cavity. Specifically, to ensure that a strong negative pressure was created in the thoracic cavity, we performed sustained inspiration efforts for 5 s (sustained method) and repeated them five times (repetitive method) in sets of 10 daily, aiming to create a definite negative pressure. Vacuum swallowing was achieved by instructing the patient to perform an inspiratory effort in conjunction with laryngeal elevation (closed glottis). The pulmonary function test results were a vital capacity of 3.63 mL, a forced expiratory volume in 1 s of 2.89 mL, and a forced vital capacity of 3.54 mL, indicating severe ventilatory limitation. Therefore, shoulder elevation to expand the thoracic cavity was recommended as a compensatory method for the decreased respiratory muscle strength.

On day 43, after PAP administration, VF showed improved oral transit without cervical neck extension using PAP. Pharyngeal clearance during swallowing improved with vacuum swallowing compared to non-vacuum swallowing. Consequently, meal duration was reduced to 40 min with a combination of balloon dilatation, PAP use, and vacuum swallowing for every three or four mouthfuls for oropharyngeal residue removal. Moreover, the patient was able to swallow saliva through vacuum swallowing, resulting in decreased expectoration. Physical examination and nutritional assessment revealed improved nutritional status with a weight of 55.9 kg, a BMI of 20.53, a serum albumin level of 4.3 g/dl, and a GNRI of 102 (no nutritional risk). His FILS remained unchanged at level eight, but dysphagia screening showed a slight improvement with an RSST of nine counts/30 seconds and a MASA score of 142 (dysphagia: moderate, aspiration: moderate).

Manometric study

Pressure and timing data were extracted using ManoScanTM software (Medtronic plc., Dublin, Ireland). Vacuum swallowing is defined as the simultaneous generation of a robust negative esophageal pressure during swallowing. Several parameters, such as the pharyngeal contractile integral (PhCI), minimum esophageal pressure (esophageal Pmin), and maximum lower esophageal sphincter (LES) pressure (LES Pmax) during swallowing, have been recorded [2,14,15]. The PhCI serves as a comprehensive measure of pharyngeal contractile force within a space-time box on the pressure topography plot extending from the velopharynx to the upper margin of the UES. The PhCI is the mean pressure within this box multiplied by the duration (s) and length (cm), expressed in units of mmHg s-cm. Esophageal Pmin and PhCI were analyzed using an independent t-test with seven swallows measured and p<0.05 as the significance level. The LES Pmax was measured in three swallows, and no significance difference test was performed.

**Figure 1:**
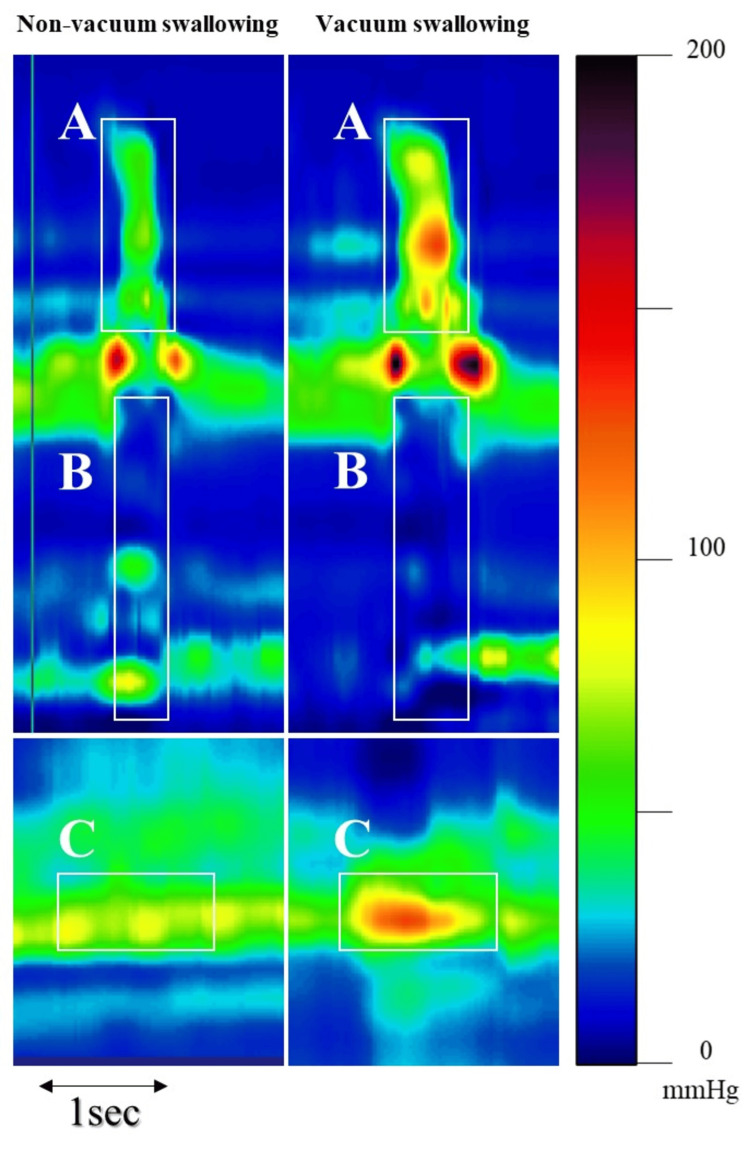
Patient's pressure topography Non-vacuum swallowing (left) and vacuum swallowing (right) are displayed. The y-axis represents the position of the catheter, and the x-axis represents time. Pressure is displayed using a color scale. (A) PhCI: pharyngeal contractile integral; (B) Esophageal Pmin: minimum esophageal pressure; (C) LES Pmax: maximum lower esophageal sphincter pressure

On day 82 of admission, the mean and standard deviation (SD) of each high-resolution manometric parameter were estimated (Table 1, Figure 1).

**Table 1:** Comparison of non-vacuum and vacuum swallowing on the HRM HRM: high-resolution manometry; PhCI: pharyngeal contractile integral; Esophageal Pmin: minimum esophageal pressure; LES Pmax: maximum lower esophageal sphincter pressure

	Non-vacuum swallowing	Vacuum swallowing	p-value
PhCI (mmHg-s-cm)	33.9±16.6	122.6±21.4	<0.001
Esophageal Pmin (mmHg)	-5.1±1.9	-14.1±1.4	<0.001
LES Pmax (mmHg)	83.9±12.5	140.2±18	Significance test not performed

The PhCI in non-vacuum swallowing was 33.9±16.6 mmHg-s-cm, while in vacuum swallowing it increased significantly to 122.6±21.4 mmHg-s-cm (p<0.001). Non-vacuum swallowing showed an esophageal Pmin of -5.1±1.9 mmHg, whereas vacuum swallowing showed a significantly lower Pmin of -14.1±1.4 mmHg (p<0.001). The LES Pmax in non-vacuum swallowing was recorded at 83.9±12.5 mmHg, whereas vacuum swallowing showed an increased pressure at 140.2±18 mmHg.

The VF showed improved pharyngeal clearance during vacuum swallowing compared to non-vacuum swallowing (Video 1).

**Video 1:** Swallowing with non-vacuum and vacuum The videofluoroscopy (VF) showed improved pharyngeal clearance during vacuum swallowing compared to non-vacuum swallowing. This video has been captured and created by the authors.

## Discussion

This case report highlights important findings regarding vacuum swallowing. Further, successful instruction and acquisition of vacuum swallowing in patients with neuromuscular disorders have been demonstrated.

With proper instructions, the acquisition of vacuum swallowing is feasible for patients with neuromuscular disorders. In the high-resolution manometer, vacuum swallowing resulted in a significantly lower negative esophageal pressure than non-vacuum swallowing. Vacuum swallowing requires actions involving the contraction of the intercostal and diaphragm muscles to create strong negative pressure in the thoracic cavity [1]. Despite weakened respiratory muscles owing to the SMA, compensation by shoulder elevation has been observed to expand the thoracic cavity. In this case, careful instruction was instrumental in ensuring the reliable establishment of negative pressure in the thoracic cavity. The repeated practice of sustained and repetitive inspiratory efforts contributed to the acquisition of vacuum swallowing.

Vacuum swallowing may result in a secondary increase in pharyngeal pressure. The typical vacuum swallowing process involves improving the pharyngeal passage of a bolus by creating a strong negative pressure in the esophagus to suck the bolus past the pharynx [1]. In cases of LMS, vacuum swallowing leads to a decrease in negative esophageal pressure without an increase in pharyngeal pressure [2]. Effortful swallowing increases the pharyngeal pressure [16]. In this case of vacuum swallowing, there was not only a significant decrease in negative esophageal pressure but also a significant increase in pharyngeal pressure. The inspiratory effort of swallowing during vacuum swallowing potentially increases the pharyngeal pressure, resembling an effortful swallow. While the generation of negative esophageal pressure was less robust than that in LMS cases [2], the increased pharyngeal pressure resulted in a more pronounced pressure gradient between the pharynx and esophagus, aiding in the removal of pharyngeal residue.

Importantly, this is the first report of successful instruction and acquisition of vacuum swallowing in a patient with a neuromuscular disorder. The efficacy of vacuum swallowing has been mainly focused on LMS, and its indication has been considered to be bulbar-type dysphagia with weak pharyngeal contraction and failure of UES opening [1, 2]. It is crucial that patients have favorable cognitive functions to understand the teaching method. However, further research is required to confirm these indications.

## Conclusions

This report describes the successful implementation of vacuum swallowing through instructions in a patient with dysphagia secondary to SMA. High-resolution manometry revealed a significant increase in pharyngeal contractile integral and a significant decrease in esophageal pressure with vacuum swallowing, which enabled the passage of a bolus through the pharynx compared with non-vacuum swallowing. Further, vacuum swallowing may benefit individuals with weak pharyngeal contractions and impaired UES opening under various conditions.
